# Behavioral Immunity in Insects

**DOI:** 10.3390/insects3030789

**Published:** 2012-08-15

**Authors:** Jacobus C. de Roode, Thierry Lefèvre

**Affiliations:** 1 Department of Biology, Emory University, 1510 Clifton Road, Atlanta, GA 30322, USA; 2 MIVEGEC (UM1-UM2-CNRS 5290-IRD 224), Centre IRD, 911 Av. Agropolis–BP 64501, Montpellier 34394, France; E-Mail: thierry.lefevre@ird.fr

**Keywords:** behavior, immunity, host-parasite interactions, qualitative/quantitative resistance, tolerance, avoidance, medication, virulence, local adaptation

## Abstract

Parasites can dramatically reduce the fitness of their hosts, and natural selection should favor defense mechanisms that can protect hosts against disease. Much work has focused on understanding genetic and physiological immunity against parasites, but hosts can also use behaviors to avoid infection, reduce parasite growth or alleviate disease symptoms. It is increasingly recognized that such behaviors are common in insects, providing strong protection against parasites and parasitoids. We review the current evidence for behavioral immunity in insects, present a framework for investigating such behavior, and emphasize that behavioral immunity may act through indirect rather than direct fitness benefits. We also discuss the implications for host-parasite co-evolution, local adaptation, and the evolution of non-behavioral physiological immune systems. Finally, we argue that the study of behavioral immunity in insects has much to offer for investigations in vertebrates, in which this topic has traditionally been studied.

## 1. Introduction

Parasites (broadly defined to include viruses, bacteria, protozoans, helminths and arthropods) pose major threats to the fitness of their hosts and natural selection should favor hosts that can effectively protect themselves against their parasites. Traditionally, most studies on anti-parasite defenses have focused on physiological and genetic mechanisms that prevent or reduce infection. In insects, these mechanisms include phagocytosis, melanization, encapsulation, coagulation and production of antimicrobial peptides [1,2]. However, hosts can employ alternative defense mechanisms, including morphological barriers [3], altered behaviors [4], changes in life-history traits [5] and symbiont-mediated defenses [6]. Although most researchers still associate the use of the word “immunity” with the physiological and genetic pathways that underlie the innate and adaptive immune systems, it is now clear that the concept of immunity needs to be extended to these non-immunological defense mechanisms to obtain a complete understanding of how hosts defend themselves against parasites [7]. 

One of the most appealing ways in which hosts can protect themselves against parasitism is through the use of altered behaviors, a series of mechanisms that we will refer to as behavioral immunity. This concept may evoke images of large-brained chimpanzees seeking out medicinal herbs with which to treat their diseases, or of cows avoiding pastures that are littered with feces and worm eggs. Indeed, behavioral immunity has received increasing attention in the study of vertebrate behavior, in which the specific mechanisms related to the perception, cognition and emotion of disease status and disease risks have been particularly well described [8], and several reviews have now been written [9,10]. In contrast, the role of behavioral immunity in insects is much less appreciated [4,11]. This is surprising, since insects provide a wide variety of innate behaviors that protect themselves or their kin against the negative effects of parasites. Moreover, as we will argue, because insects are more easily used in manipulative experiments, they provide more suitable systems to study the ecology and evolution of behavioral immunity than their large-brained and warm-blooded cousins, for which manipulative experiments are commonly unethical. Although behaviors are determined by genes and physiology, we will draw a distinction between behavioral and physiological immunity, and define the latter to strictly include non-behavioral defenses such as phagocytosis, melanization, encapsulation, coagulation and production of antimicrobial peptides. 

The scope of this review is to provide an up to date appraisal on the various anti-parasite behaviors of insects reported in a rising number of studies. In reviewing this growing field of insect behavioral immunity, we will categorize anti-parasitic behaviors into three major defense mechanisms: (1) qualitative resistance (also known as anti-infection resistance or avoidance), which prevents the establishment of parasite infection or reduces the infective dose; (2) quantitative resistance (also known as anti-growth resistance or clearance), which reduces parasite growth or parasite burdens in already infected hosts; and (3) tolerance, which does not reduce parasite infection or growth, but instead alleviates the fitness reductions caused by infection. These defense strategies all function to preserve host fitness, but their consequences on parasites are different. In particular, qualitative/quantitative resistance reduces parasite fitness, while tolerance does not. As a result, it is often expected that resistance and tolerance will differentially affect host-parasite coevolutionary dynamics, with host populations maintaining genetic variation in resistance, but not in tolerance [12,13,14,15,16].

In this review, we will categorize insect behaviors as behavioral immunity, regardless of whether they increase the direct or indirect fitness of the individuals displaying the behaviors. Although this view is often overlooked in studies on behavioral responses to diseases [17], the field of social immunity in insects has recently received interest [18,19,20,21,22], and has clearly demonstrated that individuals can increase their inclusive fitness by protecting themselves (behavioral self-immunity), their offspring (behavioral trans-generational immunity) or other relatives (social immunity).

Behavioral immunity in insects is often present but frequently overlooked. In this review, we will summarize the current state of the field, demonstrating that parasites play a major role, comparable to other biotic interactions such as predation and competition, in shaping the ecology and evolution of insect behaviors. Besides providing an updated overview of insect behavioral immunity and discussing its evolutionary implications, our goal here is also to inspire future empirical and theoretical studies in this area. We will discuss the implications of behavioral immunity on the evolution of canonical immunity, parasite virulence, host-parasite co-evolution and speciation. Finally, we hope to demonstrate that insects have a lot to offer for the study of behavioral immunity in vertebrates; although insects do not possess the cognitive skills and learning abilities of many vertebrates, they do enable researchers to carry out manipulative experiments to determine whether observed behaviors are truly involved in the fight against parasites. 

## 2. Qualitative Resistance: Avoiding Infection

**Figure 1:**
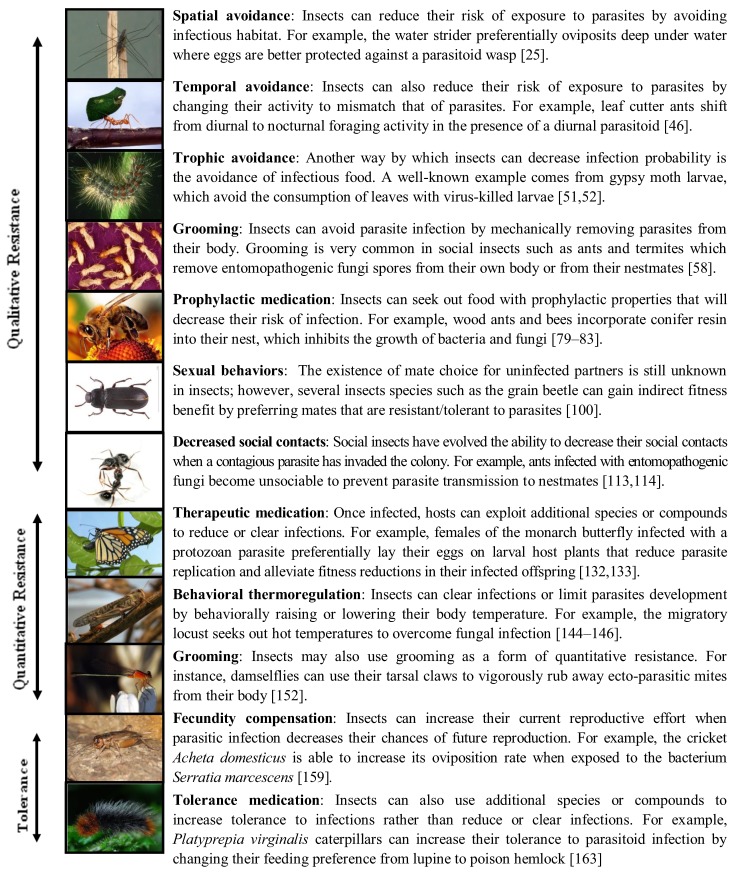
Examples of host behaviors that confer qualitative resistance, quantitative resistance and tolerance. *All pictures downloaded from wikipedia except photos illustrating spatial avoidance, decreased social contacts, therapeutic medication and tolerance medication respectively by Hiroyuki Hirayama, Volker Nehring, Jacobus C. de Roode and Virgiliu Marius Aurelian*.

Perhaps the best protection against parasites is to avoid becoming infected, and there are many behaviors by which insects can obtain such qualitative resistance. Qualitative resistance mechanisms include: (i) spatial avoidance; (ii) temporal avoidance; (iii) selective foraging (trophic avoidance and prophylactic medication); (iv) hygienic behaviors; (v) altered mating behaviors; and (vi) decreased social contacts (Figure 1). Parasite avoidance is perhaps the most documented form of behavioral defense in insects, but the underlying mechanisms through which insects perceive and respond to the presence of parasites remain elusive in the vast majority of cases.

### 2.1. Spatial Avoidance

Identifying the ultimate causes of the spatial distribution of organisms is a major goal of evolutionary ecology. Hypotheses typically invoke the selection of optimal abiotic environments (temperature, humidity), resource availability and avoidance of predation and competition. Population ecology theory suggests that, generally speaking, animal movement favors disease transmission both within and between species, as has for example been observed for West Nile virus [23]. However, animal movement may also have the opposite effect by reducing the encounter probability between hosts and parasites [24]. As such, the avoidance of parasite transmission may be a strong determinant of animal movement and habitat use.

Spatial avoidance of parasites can happen on both large and small scales. Water striders (*Aquarius paludum insularis*), for example, tend to oviposit deep under water to avoid infection with the egg parasitoid wasp *Tiphodytes gerriphagus *[25]. On larger scales, insects may migrate long distances to move away from habitats in which infective spores have built up over time [26]. Research on the monarch butterfly (*Danaus plexippus*), for example, has indicated that long-term migration enables butterflies to leave behind parasite-infested host plant habitats, and to recolonize parasite-free habitats in the spring [27]. Moreover, long-term migration also reduces parasite prevalence by weeding out infected individuals, which have reduced flight ability [27,28]. However, it is as yet unclear whether migration evolved as a mechanism to avoid parasites, or whether the reductions in parasite risk are simply a beneficial ecological consequence of the migratory behavior. 

Spatial avoidance of parasites is especially prevalent in the context of female oviposition behavior. In many insects, females crucially and directly affect their offspring’s fitness by determining where to lay their eggs, and there should be strong selection for females to lay their eggs in habitats that result in high offspring performance [29,30]. As one example, female mosquitoes are able to detect and avoid waters containing mosquito larvae infected with the trematode parasite *Plagiorchis elegans, *and thereby reduce the risk of infection for their offspring [31,32,33,34]. Similarly, studies on herbivorous insects have shown that ovipositing females may preferentially lay their eggs on plants that reduce the risk of parasitoid infection [35,36,37,38]. For example, although the cotton moth (*Spodoptera littoralis*) performs better in terms of larval and pupal weight and development time on cotton than on alfalfa plants, it strongly prefers to oviposit on alfalfa in both field and laboratory conditions [38]. This can be explained by a higher infection rate by the parasitoid wasp *Chelonus inanitus* on eggs deposited on cotton than on alfalfa [38]. Parasitoids exploit chemical cues emitted by plants to locate their host [39] and it is likely that volatiles emitted by cotton are stronger attracting stimuli for the parasitoid than those of alfalfa [38]. 

The selection of enemy-free space can take surprising forms. The seed beetle (*Mimosestes amicus*), for example, has evolved a defense whereby females protect one of their eggs from parasitoid attacks by laying inviable protective shield eggs on top [40] (see also [41] for examples of fecal shields). As another example, the golden egg bug (*Phyllomorpha laciniata*) lays eggs on conspecifics instead of host plants, turning these conspecifics into babysitting bodyguards, and resulting in greater egg survival [42,43]. Moreover, female bugs also decreased their oviposition rate in the presence of the parasitoid [42], a plastic behavior that presumably limits exposure of offspring to infection.

### 2.2. Temporal Avoidance

Parasitism not only influences the spatial distribution of insect hosts but can also affect their activity rhythms. Most evidence for temporal avoidance comes from field and experimental studies on ants and their phorid parasitoids [44,45,46,47,48]. Females of these parasitoids lay their eggs in foraging workers. Larval development occurs in the head capsule of hosts and eventually results in their decapitation. Host ant species have evolved a variety of specialized behaviors to defend workers against ovipositing parasitoids including alteration of the foraging cycle. For example, in the termitophagous ant *Pheidole titanus,* workers forage during the day in the dry season. However, during the wet season, the ants shift their foraging activity and prey on termites at night when the parasitoid fly is inactive [44]. An analogous pattern occurs in leaf-cutter ants (*Atta cephalotes*), which shift from diurnal to nocturnal activity in the presence of the diurnal parasitoid fly *Neodohrniphora curvinervis *[46].

### 2.3. Trophic Avoidance

Many parasite species are trophically transmitted, and host behaviors that reduce contact with infective stages of parasites should be favored by natural selection [49,50]. A well-known example in insects comes from gypsy moth larvae, which avoid the consumption of leaves with virus-killed larvae [51,52]. Interestingly, larvae also avoid uninfected cadavers or molasses-smeared leaf discs [51], suggesting that cues used by the host to detect and avoid contaminated patches are not specific to the virus. This non-specific avoidance resembles the selection of feces-free patches by herbivorous mammals [53] and the avoidance of cannibalism observed in many carnivorous and omnivorous vertebrates [17]. In these examples, the cues responsible for the detection are emitted by objects (dead conspecifics, feces) that are sometimes, but not always, associated with parasites. A noticeable counter example is, however, given by the infective mycelia of the fungus *Metharizhium anisopliae* that can deter both scarab beetle adults and larvae from feeding [54].

### 2.4. Grooming

Grooming is among the most frequent behavioral patterns observed in vertebrates–rats, for example, can devote up to one third of their active time to grooming [17]–but is also common in insects. Such grooming can be an effective defense against parasite infection. For example, when infective juveniles of the nematode *Heterorhabditis bacteriophora* attack the cuticle of Japanese beetle larvae, the latter fight the parasites off by brushing with the legs and rubbing with the abrasive raster, and in this way remove more than sixty percent of attacking nematodes from their cuticle [55]. Grooming behavior is especially prevalent in social insects, which often groom their relatives (allo-grooming), or the hive as a whole (social grooming). 

Allo-grooming in social insects can cause disease resistance in at least two distinct ways. First, it is a direct efficient way of mechanically removing infective parasites such as fungal conidia from nestmates before the disease spreads into the nest [56,57,58,59]. Second, allo-grooming (along with other social contacts) can indirectly boost the host’s physiological resistance to parasites [60,61]. Some of the most detailed studies on mechanical allo-grooming have been done on the termite *Coptotermes formosanus*, which is often infected with fungal pathogens. Termites preferentially groom infected nestmates more than uninfected nestmates, and can successfully remove more than 80% of the infectious fungal conidia from the cuticle of infected individuals [58]. Allo-groomers then dispose the conidia through their alimentary tracts where the fungus seems to be inhibited [58]. Inoculated termites are also often attacked and eaten by their uninoculated nestmates. The preferential grooming of infected over uninfected nestmates is most likely mediated through odors, since termites seem able to sense volatile substances emitted from fungal conidia on the cuticle of their nestmates [62,63,64]. Termites also provide an example of the second way in which allo-grooming can provide protection: the interaction between immunized and naive nestmates can improve the resistance of individuals that have not been directly exposed to a fungal parasite [60]. This social facilitation of disease physiological resistance through transfer of immune effectors (antipathogenic proteins) from exposed to naive nestmates has also been observed in ants [61], but the mechanisms underlying this phenomenon remain to be determined. 

Ants, bees and termites may also engage in social grooming (or hygienic or antiseptic behaviors), whereby workers remove parasitized brood from the nest before the disease spreads in the colony [20]. The detection and removal of diseased larvae in honeybees from the nest (e.g., [65,66,67]) has been recognized since the late 1930s [68] and can be directed against *Ascosphaera apis* (the fungal agent of chalkbrood disease [69]), *Paenebacillus* (the bacterium that causes the deadly American foulbrood [70]), and the parasitic mite *Varroa destructor* [71]. Note that we have classified these grooming behaviors as qualitative resistance because they reduce the infection probability of individual insects. However, viewed from the colony as a whole, many of these behaviors would be more appropriate classified as quantitative resistance, by reducing parasite burdens and growth in the insect colony.

### 2.5. Prophylactic Medication

As described above, insects can reduce the risk of infection by actively avoiding food that is associated with parasites. On the other hand, insects may actually seek out food that will decrease their risk of infection, for example by increasing their physiological immuno-competence [72,73] or by rendering their internal environment inhospitable to parasites [49]. Plants are good candidates for such prophylactic foods as they often vary in their nutritional qualities and also contain various chemicals that are toxic, including oxalic acid, cyanide, cardiac glycosides, alkaloids, terpenoids and tannins [74,75]. Prophylactic medication—in contrast with therapeutic medication (see Section 3.1)—is a form of medication that is displayed by both infected and uninfected individuals, and is thought to have evolved under parasite pressure. 

As with many avoidance behaviors, prophylactic medication can be directed at the individual itself or at its offspring and other relatives. Evidence for the former is currently indirect, and comes from generalist arctiid caterpillars (*Grammia geneura *[*=Grammia incorrupta*] and *Estigmene acrea*), which are frequently attacked by parasitoid flies. In the wild, both infected and uninfected caterpillars prefer poor-quality toxic plants or mixed diets (that include toxic plants) over high-quality non-toxic species, and laboratory experiments have shown that by including toxic plants in their diet, arctiid caterpillars enhance their survival in the presence of parasitoids at the expense of reduced growth efficiency [76,77,78]. Evidence for non-self prophylactic medication has been well documented in wood ants, which incorporate conifer resin pieces into their nests. Initial studies showed that resin decreases the density of bacteria and fungi in nest material and inhibits the growth of bacteria *in vitro* [79]. It was then shown that adult workers and larvae experimentally infected with the bacterium *Pseudomonas fluorescens* and the fungal entomopathogen *Metarhizium anisopliae* survived better in the presence than absence of resin [80]. Finally, in field cafeteria tests, both infected and uninfected ants showed a strong preference for incorporating resin into their nests instead of twigs and stones [81]. The use of resin has also been shown in honeybees, which commonly incorporate resinous mixtures (also known as propolis) into their nests [82,83,84]. Thus, the utilization of resin has independently evolved in multiple lineages, and strongly suggests that the incorporation of resin is a true host adaptation that evolved to combat parasites.

### 2.6. Sexual Behaviors

Contagious sexually transmitted diseases are common in natural populations and hosts should strongly prefer healthy uninfected partners to avoid infection during mating [85]. In addition to this direct fitness benefit, an indirect fitness benefit can be gained when the preference for a healthy mate is associated with heritable resistance/tolerance to parasites that will be passed to offspring [86]. Although direct and indirect fitness benefits of mate choice for uninfected partners have been thoroughly documented in vertebrate hosts [17,87,88,89], research on parasite-mediated sexual selection in insects has been lagging behind [90].

To our knowledge four studies have tested whether insects can avoid mating with infected individuals [91,92,93,94]. For example, in the milkweed leaf beetle (*Labidomera clivicollis*)*,* females spent the same amount of time and had the same number of contacts with uninfected males and males infected with the mite *Chrysomelobia labidomera*. Similarly, when males were given a choice, they simply mounted the first female contacted in most cases [91]. Likewise, Webberley *et al*. [92] found that neither males nor females of the two-spotted ladybird (*Adalia bipunctata*) were able to avoid mates infected with the sexually transmitted mite *Coccipolipus hippodamiae*. Together these studies provide no support for the parasite transmission avoidance hypothesis [85]. One potential explanation for this is that contagious sexually transmitted parasites have evolved to remain cryptic [95] and even manipulate host sexual activity to increase transmission opportunities [87] (see also Section 5.7).

Attempts to demonstrate indirect fitness benefits of mate choice for immunocompetent partners have been more fruitful, but also inconsistent. Most of them have focused on the relationship between male encapsulation responses to a foreign object and the expression of sexually selected traits. For example, wing pigmentation in damselflies [96,97,98], courtship song in field crickets [99] and pheromones of mealworm beetles [100] all showed positive correlations between female preference and male encapsulation responses. In contrast, calling songs in the house cricket *Acheta domesticus* [101] and the Australian cricket *Teleogryllus oceanicus* [102] did not correlate with males’ encapsulation response. Collectively, these results call for more research in this area, caution against the use of a single measure of immune response to reflect partners’ quality, and also caution against experiments that are based on measuring immune responses against foreign objects instead of real parasite challenge [102].

Instead of signaling immune-competence, sexual signals may also increase the risk of infection, and when that is the case, males with less conspicuous sexual signals may actually be selected for. As one example, *Gryllus integer* cricket males vary genetically in their calling songs: some males call regularly and are very successful at attracting females, whereas other males call infrequently, or not at all [103]. These latter males have evolved an alternative mating strategy whereby they intercept females attracted by calling males; although they seem to obtain less mating events than calling males, they are also less likely to be infected with acoustically orienting parasitoid flies [103] (see also [104,105,106,107,108,109,110] for a detailed example in the Australian cricket *Teleogryllus oceanicus*).

### 2.7. Decreased Contact with Conspecifics

As with sexual interactions, non-sexual social interactions increase the risk of contracting contagious parasites [24], and behaviors that allow individuals to avoid such contagious contacts should be selected for. For example, workers of the ant *Formica rufa,* which typically feed on nestmate carcasses, can discriminate and specifically avoid fungus-killed nestmates [111]. This avoidance behavior is quite specific as it is only expressed when the carcasses are covered with conidia, *i.e.*, the infectious spores of the fungus. In contrast, when carcasses host the fungal immature stages, the ants readily use them for food.

Inclusive-fitness theory [112] predicts that sick individuals should also avoid transmitting parasites to other group members if they are related, and thus quarantine themselves. Recent studies have indeed demonstrated that ants infected with the generalist entomopathogenic fungus *Metarrhizium *reduce social behaviors such as trophallaxis and grooming, spend less time with the brood, and abandon their nests to die in isolation. This nest desertion likely acts to reduce the risk of transmitting diseases to relatives [113,114] (see also [115] for similar observations in uninfected but health-compromised honey bees). 

## 3. Quantitative Resistance: Reducing Parasite Growth and Clearance

Insects do not always manage to avoid parasites and in many cases do become infected. When that happens, reducing parasite growth or clearing infection altogether may result in lower fitness loss. Insects indeed display a wide range of quantitative resistance mechanisms, including: (i) therapeutic medication; (ii) behavioral thermo-regulation; and (iii) grooming (Figure 1). 

### 3.1. Therapeutic Medication

Therapeutic medication can be defined as a series of behaviors through which infected hosts exploit additional species or compounds to reduce or clear infections, whether mediated through defensive or nutritional properties [72,116,117]. As with prophylactic medication, therapeutic medication may be directed at the infected individual itself, at its offspring, or at other relatives. The major distinction with prophylactic medication—which is displayed by both infected and uninfected individuals—is that therapeutic medication is used solely by infected individuals. Thus, prophylactic medication is a fixed response, while therapeutic medication is a plastic response, used only when individuals are infected.

Most evidence for animal therapeutic self-medication comes from correlative field studies mostly in primates in which an observed behavior (e.g., a chimpanzee eating a medicinal plant), is assumed to be beneficial to the host, without actually measuring host or parasite fitness [117]. Therefore, it has been argued that manipulative experiments are seriously needed to clearly test for the existence of animal self-medication [117,118], and insects provide suitable systems for such experiments. For example, woolly bear caterpillars (*Grammia incorrupta*) increase their taste for specific toxins, such as alkaloids contained in larval food plants when they are parasitized [119,120] and controlled experiments showed that the increased intake of toxic plants by parasitoid-infected individuals resulted in higher caterpillar survival, suggesting that this feeding change is indeed a form of therapeutic self-medication [120,121]. Similarly, careful experimentation has shown that fruit flies can use alcohol as a medicine against their parasitoid wasps: in particular, wasp-infected fruit fly larvae preferentially consumed high-ethanol fly food, which increased their blood ethanol levels, killed their infecting wasps, and increased their survival [122]. 

Therapeutic self-medication does not necessarily rely on the consumption of toxic substances, but may also be mediated through the interaction between nutrition and physiological immune responses [72,73]. For instance, *Spodoptera* caterpillars, in response to viral or bacterial infection, are able to offset protein costs of pathogen resistance by self-regulating their nutritional intake [123,124]. Similarly, infected fruit flies appear to be able to preferentially consume yeast species that equip them with a greater ability to encapsulate parasitoid eggs [125].

In some cases, insects may not be able to use medication to cure themselves, but use medication to cure their offspring instead. As one example, monarch butterflies are commonly infected with the protozoan *Ophryocystis elektroscirrha*, a detrimental parasite that reduces monarch butterfly pre-adult survival as well as adult fecundity, longevity and flight ability [28,126,127,128]. Monarch butterflies use milkweed species as their larval food plants, and several studies have shown that some milkweeds are medicinal, reducing parasite growth and increasing infected monarch longevity [129,130]. These medicinal properties are probably caused by anti-parasitic milkweed secondary chemicals known as cardenolides [131]. In a series of manipulative experiments, it was shown that infected larvae did not preferentially consume medicinal milkweed when given a choice between medicinal and non-medicinal milkweed; instead, infected females preferentially laid their eggs on medicinal milkweeds when given a choice [132,133]. This preferential oviposition results in reduced parasite growth in the offspring, to which parasites are unavoidably transmitted during the egg-laying event.

### 3.2. Behavioral Thermo-Regulation

Instead of consuming anti-parasitic food, insects can also seek out warm locations that increase their body temperature to levels that are detrimental to their parasites [134,135,136]. This behavioral fever is costly for uninfected individuals but results in suppression of parasite development in infected hosts. Behavioral fever has been observed in a wide range of insect-parasite systems, including house flies parasitized with various fungi [137,138], grasshoppers infected with microsporidian or fungal parasites [139,140,141,142,143], locusts infected with fungi [144,145,146], and crickets infected with rickettsia [147,148]. Behavioral fever can be quite specific: for example, although crickets select higher temperatures when infected with the prokaryotic parasite *Rickettsiella grylli *they do not change their thermo-preference when exposed to a bacterium (*Serratia marcescens*), a protozoan gut parasite, or a tachinid fly parasitoid (*Ormia ochracea*) [148]. Finally, elevated temperatures can be obtained by social behaviors instead of individuals seeking out warmer environments: in particular, honeybees communally raise the temperature of their hive in response to an infection with the heat-sensitive pathogen that causes chalkbrood [149]. 

Behavioral thermo-regulation can also take the form of chilling whereby sick individuals seek out lower instead of higher temperatures. As one example, field observations on bumblebees indicated that conopid fly-infected bumblebees spend the night at low outside temperatures instead of entering the warmer hive [150]. Choice experiments confirmed that infected workers preferentially spend time in cold areas and that this behavior reduced the chances of successful parasite development and hence increases bumblebee survival [150]. Similarly, acanthocephalan-infected cockroaches spend enough time in cool temperatures to severely retard the development of their parasites [151]. 

### 3.3. Grooming

Therapeutic medication and behavioral thermo-regulation are indirect defense mechanisms, through which hosts employ environmental factors (food and temperature) to reduce their parasite burdens. However, hosts may also actively reduce parasite numbers by grooming. As described in Section 2.4, grooming is most often used as a way to reduce the probability of infection (*i.e.*, qualitative resistance). However, grooming may also be used as a form of quantitative resistance. For instance, the damselfly *Ischnura verticalis* uses its tarsal claws to vigorously rub away ecto-parasitic mites from its body [152]. 

## 4. Tolerance

When hosts cannot avoid infection (qualitative resistance), or reduce parasite growth once infection has occurred (quantitative resistance), they can still limit the negative effects of parasitism through tolerance mechanisms. Tolerance can be obtained through alterations in life history, whereby hosts compensate for negative fitness consequences by investing heavily in producing offspring (fecundity compensation). Alternatively, tolerance can be obtained by feeding on diets that allow hosts to maintain health despite parasite infection (Figure 1). 

### 4.1. Fecundity Compensation

When future reproductive opportunities of an individual decline, life history theory predicts an increase in current reproductive investment in iteroparous organisms [153,154]. Parasites, in reducing their host’s longevity and/or fecundity, may trigger such changes; and it is therefore expected that hosts close to death or castration should enhance their current reproductive effort at the expense of future reproduction [155,156,157]. This fecundity compensation or terminal investment has been observed in insects infected with parasitic mites [91,158], bacteria [159,160] and microsporidians [161]. This plastic adjustment of reproductive effort in infected insect hosts can be quite specific: although exposure to the bacterium *Serratia marcescens* or lipopolysaccharides (components of bacterial cell wall) induced an increased rate of egg laying in crickets, infection with the larvae of the parasitoid fly *Ormia ochracea* or injection of sephadex beads had no effect [159].

### 4.2. Tolerance Medication

Apart from fecundity compensation, hosts may also obtain tolerance by preferentially consuming food that allows them to maintain health and fitness despite parasite infection. Most of the examples on medication in insects as described above have been viewed in terms of obtaining qualitative or quantitative resistance. However, a recent study has demonstrated that the consumption of particular species of milkweed by parasite-infected monarch butterflies can also enhance tolerance. In particular, when infected monarchs were reared on milkweeds with high concentrations of cardenolides, they experienced a lower reduction of lifespan for a given level of parasitism [162]. These results suggest that the observed preferential oviposition of infected monarchs on high-cardenolide milkweed [132,133] does not only provide these monarchs’ offspring with greater quantitative resistance, but also with greater tolerance. 

The provision of tolerance may also explain the preferential consumption of poison hemlock by parasitoid-infected *Platyprepia virginalis* caterpillars. In particular, hosts infected with the parasitoid fly *Thelaira americana* change their feeding preference from lupine to hemlock, but this does not result in increased resistance. Instead, both host and parasite appear to benefit from the altered feeding preference, with hosts surviving the emergence of the parasitoid, and parasitoids obtaining a higher pupal mass [163]. Overall, these results are consistent with theoretical work that suggests that tolerance maintains host fitness without reducing that of the parasite.

## 5. Ecological and Evolutionary Consequences

### 5.1. Costs and Trade-Offs

As described in the previous sections, hosts have evolved a wide range of behaviors that protect them against the negative effects of parasites. Based on this review, we have no doubt that behavioral immunity is a potent and crucial defense mechanism in nature, yet behavioral immunity is still ignored in most studies on host-parasite interactions. This neglect has at least two negative consequences. 

First, without considering behavioral immunity, important insights into the nature of a host-parasite interaction may be lost. For example, we may predict that individuals living in groups should invest more in defense mechanisms because of increased risks of infection [164]. If we focus our experiments entirely on non-behavioral immune mechanisms, we may erroneously conclude that there is no effect of group living on immunity, when in fact individuals display behavioral defenses, such as social grooming. Furthermore, researchers may compare parasite burdens between different host genotypes. If they find no variation in parasite growth, they may mistakenly conclude that hosts do not vary in parasite defense, even though hosts actually vary in the degree to which they compensate for parasite-induced fitness loss through fecundity compensation. Thus, the study of behavioral immunity–in addition to other forms of defense–can provide important insights into the interactions between hosts and their parasites. 

Second, the study of behavioral immunity can provide major insights into the costs of immunity and its trade-offs with other life-history traits. It is generally expected that host defense is costly [164,165,166,167], and many studies have indeed found trade-offs between resistance and life history traits such as longevity, reproduction, developmental time and competitive ability [168,169,170,171,172]. However, not all studies have found an obvious cost of resistance, e.g., [173,174], and we suggest that this may be partly due to the neglect of behavioral defenses. For instance, if a behavioral defense is the main line of protection against a parasite, then we may not expect great investments in non-behavioral defenses, and hence we may not expect to easily detect costs associated with such non-major defenses. Alternatively, the costs of immunity may be found in other immune responses, instead of life-history traits such as longevity or body weight. For example, the ability to mount a strong non-behavioral response may come at the cost of a reduced ability to mount a behavioral response.

The assertion that behavioral immunity may trade off with non-behavioral defenses implicitly assumes that behavioral immunity, like other defenses, is costly (but see [175]). Although few studies have investigated such costs, the emerging picture is that behavioral defenses are costly indeed [17,22,176]. For example, oviposition preference for deep sites by water striders can result in increased egg mortality (see [177] and Section 5.3); production of protective egg shields is energetically costly and can result in a reduced lifetime reproductive output [40]; and behavioral fever in locusts can result in a decreased growth rate of the host [139]. Although behavioral and non-behavioral defenses both carry costs, the type of costs between these defenses may differ. In particular, non-behavioral responses may carry more energetic costs, while behavioral responses may more often carry opportunity costs: for example, mounting a strong cellular response may result in decreased energy for reproduction, while behavioral avoidance of conspecifics may result in a direct lack of reproduction opportunities. 

### 5.2. Loss or Reduction of Non-Behavioral Immunity

As described here and elsewhere [7], hosts have evolved a plethora of mechanisms to defend themselves against parasites. Although an intuitive prediction may be that hosts should employ as many simultaneous defenses as possible, evolutionary theory predicts that this is not the case. Indeed, because defense mechanisms are costly, a general expectation is that organisms should invest in only a subset of defense mechanisms [165,167]. However, there is still a lack of studies supporting this prediction, and one potential reason for this is that behavioral defenses have been largely ignored in the study of trade-offs between alternative defenses. This is unfortunate, because host organisms that employ behavioral defense mechanisms provide ideal opportunities to study the evolution of immunity and the trade-offs between different defense mechanisms. This is because the behavioral avoidance or treatment of infection renders the use of a non-behavioral physiological immune system superfluous, and may therefore result in a lack of such immunity–either as a fixed or plastic response. 

Currently, indirect evidence for this stems from studies on honey bees and wood ants. Honeybees lack many of the canonical immune genes that other insects possess in their genome, and it has been suggested that this may be a result of the employment of alternative defense mechanisms [178]. In particular, as described above, honeybees display a range of social behaviors to reduce infection risk [20,82]. Moreover, wood ants are well known to incorporate anti-microbial resin in their nests [79,80,81], and the presence of resin has been shown to reduce plastic investment in physiological anti-microbial activity [179]. These results are consistent, if not conclusive, with the hypothesis that the use of behavioral defenses has resulted in reduced investments in canonical physiological immunity.

Due to the universal expectation that immunity is a costly trait, we predict that behavioral defenses will commonly result in reduced investments in other forms of immunity on a macro-ecological scale. As one final example, fruit flies appear to be able to defend their offspring against parasitoid attack by reducing their egg-laying rate when parasitoid wasps are present. However, in a two-species comparison, *Drosophila melanogaster*, which has a lower level of physiological resistance, reduced its egg-laying rate more strongly than *Drosophila simulans*, which has a higher level of physiological resistance [180].

### 5.3. Parasite risk and Behavior: Plastic and Fixed Behaviors

Although it is clear that hosts are likely to evolve only a subset of possible parasite defenses, can we make any predictions about what specific defenses a host should evolve? A well-developed theoretical framework is currently lacking, but it is often argued that high risks of parasitism should select for fixed anti-parasitic defenses, while low and variable risks should select for plastic defenses. These predictions have been mostly developed in the context of prophylactic versus therapeutic self-medication, but they should equally apply to other behavioral defenses. In terms of medication, it has been hypothesized that when the risk of parasitism is low and unpredictable, hosts benefit from therapeutic self-medication (e.g., [49,81,117,181,182]), a plastic behavior by which infected individuals use anti-parasitic species/compounds which themselves are costly to uninfected individuals. In contrast, when parasitism risk is high and predictable, a fixed behavior may evolve by which all host individuals use anti-parasitic species/compounds to prevent infection or reduce the risk of heavy infections. This hypothesis is based on the assumption that induced defensive phenotypes carry costs [120,183,184], which outweigh the benefits when parasite risk is high and predictable. One way to test this hypothesis is to compare multiple host populations that vary in their risks of parasitism, and by associating the occurrence of prophylactic and therapeutic medication with the risk of parasitism. 

Many of the behavioral defenses that researchers have described so far are plastic responses. For example, the behavior by which seed beetles protect one of their eggs from parasitoids by laying inviable protective shield eggs on top appears to be plastic, with females adjusting the amount of protection they provide depending on the risks of infection: in the absence of parasitoids, females hardly lay any eggs in stacks, but when the parasitoid is around, more than half of them do so [40]. 

Key to the evolution of plasticity is the occurrence of environmental cues that an organism can use to detect parasitism, and that it can alter its behavior in response. Plasticity may also be affected by effective learning. In the water strider *Aquarius paludum*, for example, eggs oviposited deep under water suffer less from infection by a parasitoid wasp than eggs laid at the water surface or just under water [25]. In an experiment, ovipositing females were exposed to no parasitoids, a low density or a high density of parasitoids. The same females were then allowed to oviposit in parasitoid-free aquariums. Females that had experienced the wasps oviposited at a greater depth than those that had not been exposed to the parasitoid and this difference was greater when females had been exposed to the high parasitoid density [185]. Overall, although most observed defensive behaviors are plastic, not fixed, it is unlikely that this is due to a universal low risk of parasitism. Instead, we favor the hypothesis that studies are more likely to detect altered behaviors in response to parasitism than responses that have become fixed due to high parasite pressure in the past. 

The evolution of fixed behavioral immune defenses such as diet selection or habitat choice to escape parasites can result in niche expansion of insect hosts [29]. Likewise, host adaptations to novel environmental conditions can favor some level of reproductive isolation and may ultimately lead to ecological speciation and adaptive radiation in insects. For example, *Drosophila* flies that oviposit on amanitin-containing mushrooms provide protection to their offspring against parasitic nematodes [186]. This anti-parasite behavior may have selected for amanitin tolerance in mycetophagous *Drosophila* and may also contribute to the ongoing adaptive radiation observed in the *D. quinaria* species group [186,187]. As another example, larvae of the fly *Rhagoletis pomonella* feeding on apple fruits experience reduced levels of parasitoid attack compared to larvae feeding on hawthorn fruits [35]. This result has led Feder [35] to suggest that parasitoids may drive the fly habitat use and hence may contribute to the sympatric host races formation observed in this species.

### 5.4. Local Adaptation

Because the forces of natural selection vary in space and time, divergent selection should result in evolution of traits that provide an advantage under local environmental conditions [188,189,190,191]. Many empiricists and theoreticians have used host-parasite interactions to study local adaptation [189,192,193]. These interactions are ideal to study local adaptation because hosts and parasites impose strong selection on each other: parasites reduce host fitness and natural selection should strongly favor hosts that can avoid, resist or tolerate parasite infection. Likewise, natural selection should strongly favor parasites that can effectively circumvent host defenses to infect their hosts. Evolution should therefore lead to parasites that optimally infect hosts that they encounter in their local environment or hosts that optimally resist local parasites. 

As predicted by theory, many studies have found that parasites have higher fitness in their local than foreign hosts, suggesting that parasites are able to adapt to their local host genotypes, e.g., [194,195,196,197,198,199,200,201,202,203]. Other studies have found local adaptation of hosts to their parasites [204,205,206]. Surprisingly, however, the majority of studies have not found evidence for local adaptation of either hosts or parasites [192,193,205]. For instance, 30 out of the 54 local adaptation studies reviewed by Greischar and Koskella [192] did not find evidence for local adaptation. Theoretical models have provided a number of explanations for why local adaptation may not occur or may not be detected, including low parasite specificity, time-lagged coevolutionary dynamics and between-population gene flow [197,207,208,209]. 

However, a major–and often overlooked–potential reason for an observed lack of local adaptation is that most investigations on hosts and parasites focus entirely on genetic resistance mechanisms, ignoring behavioral immunity. This is problematic: if hosts have locally adapted to resist or tolerate their parasites by means of behavioral mechanisms, then ignoring such mechanisms may lead to the erroneous conclusion that hosts and parasites are not locally adapted. We therefore strongly encourage researchers to carry out local adaptation studies in which hosts are allowed to display their natural behavioral defense mechanisms, as this will most certainly increase our understanding of the local coevolutionary dynamics between hosts and parasites. 

### 5.5. Resistance *vs.* Tolerance

The critical difference between resistance and tolerance is that resistance directly reduces parasite fitness, whereas tolerance does not. As a result, theoretical models have suggested that hosts may maintain genetic polymorphisms in resistance, but not in tolerance [12,15,210]. This is because the evolution of resistance should result in a negative epidemiological feedback through which a higher frequency of resistant hosts will reduce parasite transmission and prevalence to below the level at which resistant hosts are selected for (assuming that resistance is costly); once susceptible hosts increase in frequency again, parasite transmission and prevalence will increase, resulting in selection for resistant hosts once more. In contrast, with tolerance the epidemiological feedback is positive: when the frequency of tolerant hosts increases in the population, parasite transmission and prevalence increase, causing further selection for tolerant hosts. 

Based on these predictions, we would expect to find genetic variation in behaviors that result in qualitative and quantitative resistance, but not in behaviors that provide hosts with a tolerance. A major problem with testing these predictions is that few studies on behavioral immunity actually determine whether hosts vary genetically in their behaviors. As one notable example, Parker *et al* [52] showed that different families of gypsy moth larvae exhibited various levels of avoidance of virus-killed cadavers, and concluded that there is genetic variation in host avoidance behavior. Another problem with these predictions is that the theory on tolerance is still relatively undeveloped as compared to the theory on resistance, and that further theory is likely to alter these predictions. For example, a recent study on tolerance made an explicit distinction between tolerance mechanisms that act on maintaining host fecundity rather than host lifespan [16]. The reason for this difference is that when tolerance allows hosts to maintain their lifespan despite heavy parasite infection, parasite fitness can actually be enhanced by increasing the period over which transmission can occur. This leads to a positive epidemiological feedback, resulting in a fixation of tolerance. In contrast, when hosts tolerate infection by maintaining their fecundity instead, the effect on parasites may be neutral, and variation in tolerance could evolve [16]. Thus, with regards to behavioral immunity, we may find more genetic variation in fecundity compensation than in behaviors that provide hosts with mortality tolerance.

### 5.6. Virulence Evolution

Hosts impose strong selection on their parasites through their defenses, but the host-induced selection pressures depend critically on whether hosts utilize qualitative resistance, quantitative resistance or tolerance. The reason for this differential selection, again, stems from the fact that resistance reduces parasite fitness, but tolerance does not.

Although qualitative and quantitative resistance both reduce parasite fitness, theoretical models have shown that these types of resistance may have different consequences for parasite evolution [211]. In particular, studies have suggested that quantitative resistance selects for parasites with higher intrinsic virulence [211,212,213], while qualitative resistance does not [211,213]. These models are based on the assumption that parasite evolution is driven by a trade-off between virulence and transmission, such that parasites cannot increase their transmission rate without simultaneously increasing their virulence, e.g., [214,215,216,217]. The outcome of this trade-off is the selection for parasite genotypes with intermediate levels of within-host growth, at which parasite fitness is maximized [127]. In the case of quantitative resistance, within-host parasite growth is reduced to sub-optimal levels, resulting in lower parasite fitness; as a consequence, parasites evolve higher intrinsic growth rates to overcome this reduction. Parasites selected in such a way would have higher than optimal growth in non-resistant hosts, and thereby cause higher virulence. 

In contrast, it is often argued that qualitative resistance does not directly reduce within-parasite growth and does therefore not select for parasites with increased rates of growth and virulence. This expectation is based on the assumption that anti-infection resistance is an all-or-nothing trait: parasites have a certain probability to infect a host, and when they do, the ensuing infection always results in the same growth, virulence and transmission. When this happens, anti-infection resistance may actually reduce the probability of infection, and thereby reduce the incidence of mixed-genotype parasite infections in a population. Because it is often assumed that mixed-genotype infections select for higher parasite virulence [214,216,218,219,220,221,222,223], a reduction in the occurrence of mixed infections may result in the selection for lower parasite virulence [213,220]. However, it was recently shown that qualitative resistance may also select for increased virulence: this happens when such resistance acts to reduce the infective dose of the parasite, and with that the within-host exploitation that the parasite can attain. Parasites can then also compensate by evolving a higher per-parasite growth rate, and consequently a higher intrinsic virulence [130]. 

The potential effects of tolerance on virulence evolution are currently less clear. As mentioned multiple times, the major difference between resistance and tolerance is that resistance reduces parasite fitness, while tolerance does not. This has led some authors to speculate that tolerance–in contrast with resistance-should not result in antagonistic coevolution between hosts and parasites [13], but this may not necessarily be true [224]. For example, although the evolution of tolerant hosts may result in commensal relationships between host and parasite [225], such commensalism is an expressed outcome determined by both host and parasite together. Because host tolerance reduces the cost of parasite virulence to parasites (by killing its host a parasite can reduce its transmissible period, e.g., [127,215,216,218,226,227]), parasites can in fact evolve greater intrinsic growth and virulence [225,228]. When infecting a non-tolerant host, the expressed virulence of such parasites would be higher than that before tolerance evolved [225]. 

Overall then, it appears that quantitative resistance, qualitative resistance that reduces parasite doses and tolerance may all select for parasites with higher intrinsic growth and virulence in natural populations. Although most of these studies have considered resistance as a genetic trait or as a trait conferred by vaccination, these results generalize to behavioral immunity. For example, theory has shown that the medicative use of toxic plants by parasite-infected monarch butterflies may select for more virulent parasites [130]. As one fruitful way to examine how behavioural immunity may affect parasite virulence evolution, we suggest studies in which researchers compare populations of hosts that display variable behavioural defences and compare the intrinsic virulence of the parasites that have evolved in these populations.

### 5.7. Parasites Strike Back: Manipulation

Host-parasite interactions are subject to a continuous coevolution, with hosts being under strong selection to minimize parasite-associated fitness loss, and parasites being under strong selection to maximize between-host transmission. Thus, when observing behaviors of parasite-infected hosts, it cannot be *a priori* assumed that such behaviors are host adaptations. Instead, behavioral changes can be parasite adaptations that facilitate transmission (the “parasite manipulation hypothesis” [229,230]). Alternatively they can reflect adaptations that benefit host and parasite simultaneously [231], or represent side effects of infection with no adaptive value for either host or parasite (the “boring by-product” hypothesis [229]).

The study of parasite manipulation of insect host behavior has been a very fruitful field of investigation and there are now many more examples of such behaviors than of behaviors that protect hosts against their parasites (reviewed in [4,232,233]). The fact that there are more examples of parasitic manipulation than host behavioral immunity could have a biological reason: it is generally assumed that parasites have greater evolutionary potential than their hosts because they often have larger population sizes, shorter generation times and higher rates of mutation and migration than their hosts (e.g., [196,234]). In addition, parasites have more to lose from a failure to infect (their whole fitness), than hosts lose from a failed defense (in most cases: part of their fitness, not their whole fitness). As a result, selective pressures acting on parasite transmission may be stronger than those acting on host defense (life-dinner principle [235]). However, theoretical models have shown that hosts can be ahead in the coevolutionary arm races [236,237,238], suggesting that the higher number of parasitic manipulation examples may also be the result of a study bias toward such behaviors. Indeed, as we have described in this review, behavioral host defenses against their parasites come in many forms, and it is our expectation that when these behaviors are looked for more extensively, they will be found to be very common indeed.

In other situations, both hosts and parasites benefit from the behavioral changes. This can happen when parasite infection, which decreases host fitness, triggers host compensatory responses that reduce the cost of infection, while maintaining parasite transmission [231]. Thus, when host behavioral tolerance concords with parasite success, behavioral changes can be favored by natural selection acting on host and parasite simultaneously. As one example of such a behavior, males of the beetle species *Labidomera clivicollis* increase their sexual behavior in response to infection with the lifespan-reducing ectoparasite *Chrysomelobia labidomera*. This behavior favors both the host–which maintains its fitness by increasing its mating rate–and the parasite–which obtains transmission to higher numbers of sexual partners [91]. Overall, in order to determine whether altered host behaviors are host or parasite adaptations, one needs careful experiments and a proper understanding of the life cycle of both host and parasite. 

## 6. Lessons for Vertebrates

Great apes and other vertebrates first opened our eyes to the exciting idea that animals can use medication to fight their parasites. Since those seminal studies, it has become clear that behavioral defenses extend far beyond the use of herbal medicine in vertebrates. Species as diverse as chimpanzees, ants and worms are now known to display an amazing diversity of behavioral defenses against parasites, ranging from avoiding virus-killed cadavers and modifying eggs into shields to seeking out hot temperatures and increasing mating rates. Although these studies started off with the careful observation of large primates in the field, we feel that insects have a lot to offer for the future study of behavioral immunity in a large range of animals, including vertebrates.

The major advantage of using insects for the study of behavioral immunity over vertebrates is the ability to perform manipulative experiments that are not possible or ethical with most vertebrates. For example, when studying behavioral medication in monarch butterflies, we were able to mate large numbers of butterflies in the laboratory, specifically infect some of them with virulent parasites, while leaving others uninfected. We then placed individual butterflies into a flight cage in which they had a choice between medicinal and non-medicinal food plants, and we observed preferential oviposition on the medicinal plants. In additional experiments, we reared monarch butterflies on these different food plants, and investigated mortality rates. Obviously, such manipulative experiments, in which animals are reared to directly measure their behavior and the mortality that they suffer in response to parasitism is unethical in most vertebrate species. 

Perhaps the biggest lesson that insect studies have taught us is that the types of behaviors that hosts display in response to parasitism can vary from host adaptations to parasite adaptations to adaptations that benefit both host and parasite. Thus, as we described above, simply observing an altered behavior is not sufficient to conclude that behavior is a host defense against parasitism. In fact, what insect studies have taught us is that a series of conditions has to be met to conclude that a host behavior is an anti-parasitic defense. In particular, we recommend the testing of the following conditions in the study of behavioral immunity. First, the observed behavior has to alleviate the fitness loss due to parasitism. In other words: does the behavior actually help? In the case of plastic responses, this question is relatively straightforward to ask: for example, if infected grasshoppers prefer to use hot temperatures, don’t provide them with such an environment, and they should suffer more fitness loss than if they were provided with the ability to choose a hot environment. For fixed responses, answering this question is a bit harder: how does one test whether a behavior that all individuals display actually helps against parasitism? As one approach, it may again be possible to take away the ability for hosts to display their protective behavior: for example, in the case of wood ants, artificial nests can be created in which the ants are unable to incorporate resin, and this should consequently result in more infection and fitness loss. Second, although behavioral immunity may increase parasite fitness as well as host fitness, behaviors that solely increase parasite fitness cannot be classified as behavioral immunity. For example, altered behaviors that result in increased parasite transmission, without alleviating host fitness loss, are not host adaptations. In contrast, behaviors that enhance both host and parasite fitness can be categorized as behavioral tolerance. Third, the observed behavior has to be relevant in the environment in which hosts and parasite naturally interact. Thus, it may be hard to draw firm conclusions from studies that are solely based on artificial diets that do not occur in nature. Such studies are of course useful, in that they provide us with insights into the particular chemicals and nutritional compounds that are involved in behavioral immunity, but additional studies are required to determine that in their natural environment, hosts are still able to display the protective behavior. Finally, it is important to consider inclusive fitness. Many insects display protective behaviors that do not directly alleviate their direct fitness loss, but instead protect their offspring or kin from the negative effects of parasitism. Without such an inclusive fitness approach, it is likely that many important behavioral defenses go unnoticed.

## 7. Conclusions

In this review, we have summarized behaviors that insects display to fight back against their parasites. We have categorized these behaviors into three classes of defense (qualitative resistance, quantitative resistance, tolerance), because these defenses have varying effects on the co-evolutionary dynamics between hosts and their parasites. Although the study of behavioral immunity in insects is still in its infancy, it is already clear that insects display an enormous variety of anti-parasitic behaviors, and that without considering these behaviors important insights into the interactions between hosts and parasites will be missed. We hope to have persuaded the reader that the study of behavioral immunity provides novel insights into host-parasite coevolution, the costs of immunity, the evolution of canonical physiological immunity, the evolution of parasite virulence, and the study of local adaptation. Overall, we hope that this review will inspire researchers to take a new look at their study organisms, and to consider the exciting behaviors that these organisms may have evolved in response to their parasites. 
